# Eating attitudes and depressive symptoms in a LGBTIQ sample in Turkey

**DOI:** 10.3389/fpsyt.2022.1014253

**Published:** 2022-11-11

**Authors:** Hayriye Gulec, Tayfun Torun, Aneliana da Silva Prado, Stephanie Bauer, Christine Rummel-Kluge, Elisabeth Kohls

**Affiliations:** ^1^Interdisciplinary Research Team on Internet and Society, Faculty of Social Studies, Masaryk University, Brno, Czechia; ^2^Department of Psychology, Faculty of Arts and Sciences, Bursa Uludag University, Bursa, Turkey; ^3^Department of Philosophy, Faculty of Arts and Sciences, Bursa Uludag University, Bursa, Turkey; ^4^Department of Psychiatry and Psychotherapy, Medical Faculty, Leipzig University, Leipzig, Germany; ^5^Department of Psychology, Federal University of Parana, Curitiba, Brazil; ^6^Federal Institute of Education, Science, and Technology of Parana, Curitiba, Brazil; ^7^Center for Psychotherapy Research, University Hospital Heidelberg, Heidelberg, Germany; ^8^Department of Psychiatry and Psychotherapy, University Leipzig Medical Center, Leipzig University, Leipzig, Germany

**Keywords:** eating attitudes, depressive symptoms, sexual minority, LGBT, LGBTIQ

## Abstract

**Background:**

Lesbian, gay, bisexual, transgender, intersexual and queer (LGBTIQ) individuals are often stigmatized due to their minority status. Sexual-minority stress is often discussed as a risk factor for the increased mental health problems reported in this population.

**Objective:**

The current study (1) investigated eating attitudes and depressive symptoms in a sexual minority sample from Turkey who identify themselves as LGBTIQ and (2) explored the role of sexual minority stressors beyond the potential predictors of eating attitudes and depressive symptoms in this population.

**Methods:**

Recruitment was supported and streamlined by several Turkish NGOs and LGBTIQ community networks. Sociodemographic measures, eating attitudes, depressive symptoms, sexual minority stressors (e.g., heterosexist experiences, internalized homophobia), and the potential predictors of eating attitudes and depressive symptoms were assessed with an anonymous online survey between February 2022 and June 2022. The sample consisted of 440 participants. The mean age was 31.92 (SD = 11.82). The majority of the participants reported their current gender identity as male (64.3%; *n* = 440) and their sexual orientation as attracted to men (62.8%; *n* = 439). For 79.7% of the participants, the assigned sex at birth was man (*n* = 439).

**Results:**

Two separate three-stage multiple hierarchical regression analyses were conducted, controlling for sociodemographic characteristics and the risk and protective factors of eating attitudes and depressive symptoms. Disturbed eating attitudes were predicted by assigned female sex at birth, higher scores for depression, social isolation, and the total number of heterosexist experiences, and lower distress related to heterosexist experiences. Depressive symptoms were predicted by assigned female sex at birth, lesbian sexual orientation, disturbance in eating attitudes, increases in generalized anxiety, and distress related to daily heterosexist experiences.

**Conclusion:**

The current study demonstrated the significant role of sexual minority stressors in the prediction of disturbed eating attitudes and depressive symptomatology beyond the general psychosocial vulnerability factors. These findings emphasize the need for developing strategies to reduce prejudicial attitudes at the societal level and to enhance the skills of LGBTIQ individuals in coping with sexual minority stressors in Turkey.

## Introduction

Lesbian, gay, bisexual, transgender, intersexual, and queer (LGBTIQ) individuals are often stigmatized as a sexual minority (1). Negative experiences, such as disapproval, exclusion, rejection by their families and close social circles, and discrimination in education, employment, housing, and access to social services, are not uncommon (2, 3). Accordingly, previous studies have shown a heightened risk for mental health conditions, including disordered eating behaviors and depressive symptoms in LGBTIQ individuals compared to heterosexual individuals (4, 5).

Minority stress theory is one of the major theoretical frameworks that explains the increased rates of mental health problems in sexual-minority individuals (6). It connects mental health problems in LGBTIQ individuals to stressors related to their sexual-minority status. The minority stressors may be distal (i.e., external) and proximal (i.e., internal). Distal minority stressors are negative experiences due to prejudicial attitudes in the environment, such as victimization, blaming, and harassment. Proximal minority stressors include the internalization of these prejudicial attitudes by the individual and result in self-stigma, such as the internalization of homophobic attitudes and the concealment of sexual identity. The current study examined the roles of psychosocial vulnerability factors and minority-related stressors in order to explain disturbed eating attitudes and depressive symptomatology in a sample of LGBTIQ individuals in Turkey.

Most previous research on eating disorders focused on female, cisgender, and heterosexual populations (7). Few studies that focused on sexual minorities and gender-diverse populations showed clear associations between sexual identity or orientation and eating disorders (8–12). For example, gay and bisexual secondary school boys and girls were more likely to report purging behavior and the use of diet pills than their heterosexual counterparts, who more often reported a positive body image and no indications of eating concerns (13). Another study with a large sample of adolescents in the United Kingdom found that sexual-minority girls had twice the likelihood of purging and binge eating than heterosexual girls (14).

Given that sexual-minority and gender-diverse populations are prone to experiencing negative social evaluations, interpersonal theories seem to be applicable to them. One model that incorporates interpersonal and psychological difficulties into the development of eating disorder symptoms is the “interpersonal theory of eating disorders” (15). This model highlights inadequate social situations that involve real or perceived negative evaluations by others, such as a hindered feeling of social belongingness, to explain eating disorders (16). It suggests that such interactions lead to conflicts with oneself, lower self-esteem, and cause negative affect, which trigger or maintain eating disorder symptoms, such as dieting, in order to enhance self-esteem or binge eating for emotional regulation. Another model is the “tripartite influence model” (17), which postulates that exposure to idealized appearances through media, parents, or peers causes adolescent girls to develop body dissatisfaction through the processes of internalizing ideal appearances and social appearance comparisons. Recently, this model, alongside the minority stress theory, was included in a theoretical consideration to generate a new model specific to sexual-minority women (18). The examination of the model showed similar correlates for disordered eating behaviors among heterosexual and sexual-minority women in terms of the internalization of sociocultural norms, social resources, emotion regulation, negative affect, and body surveillance. Overall, the model emphasized the intersection of the psychosocial experiences of eating disorders in general populations, with identity-related experiences, such as harassment, heterosexism, and internalized minority stress.

Studies report the significant role of general psychosocial factors in disordered eating and the overall wellbeing of LGBTIQ individuals. For example, one study found significant associations between weight-based victimization by family members and poorer self-rated health, less self-esteem, and depressive symptoms among LGBTQ adolescents (19). Adolescents who experienced weight-based victimization in their families also reported lower positive attitudes from family members toward their LGBTQ status and lower family connectedness (19). In another study, weight-based victimization from family members was related to dysfunctional eating behaviors (e.g., binge eating), dieting, or poorer weight-related health (e.g., exercise avoidance, less physical activity, higher stress levels) (20). The significant relationships remained after accounting for participants' age, BMI, sexual and/or gender identity, and race (20). These findings were supported by another study, which showed that parents could influence their daughters' disordered eating behaviors *via* body esteem, but regardless of their body-esteem LGBTQ+ girls were engaged in caloric restriction if they experienced general victimization (11).

There is evidence to indicate sexual orientation disparities in disordered eating, weight-related behaviors, and their predictors among LGBTIQ individuals. For instance, a recent study found that bisexual women and gay men reported significantly higher body weight dissatisfaction than lesbian women, bisexual men and their cisgender counterparts and the highest body weight misperception was present in gay men (21). In another study, lesbian women showed higher rates of being prone to eating disorders than gay men did (9). Also, lesbian women were more likely to report a heightened weight-based self-worth than gay men. However, a recent systematic review found that disordered eating behaviors were more pronounced during adolescence than in young adulthood for LGBTIQ females (22). Furthermore, it concluded that disordered eating and weight-related behaviors were more consistent among males than females in this population. The highest rates in dissatisfaction with eating patterns were reported for transgender and non-conforming adults. The general proneness for eating disorders was predicted by depression, perceived stigma, and self-compassion in gay men; depression in lesbian women; and self-compassion in transgender and non-conforming adults (9).

Similar to disturbed eating attitudes, sexual-minority individuals reported heightened rates of depressive symptomatology compared to heterosexual people (23). Studies demonstrated that stressors related to sexual-minority status, including victimization (24), perceived discrimination (25), and harassment (26), were associated with depression. Also, general psychosocial determinants, such as perceived social support (27), and psychological resources, such as self-esteem (28), significantly mediated the association between sexual-minority status and depressive symptomatology. Studies also found sexual orientation disparities in depressive symptomatology within LGBTIQ subgroups and indicated a substantial burden for bisexual individuals due to the higher prevalence of major depressive disorders as compared to gay and lesbian individuals (29).

Mood disorders are related to changes in metabolism and eating attitudes (30). Accordingly, studies indicate an association between depressive symptoms and unhealthier eating styles and body weight dissatisfaction (21, 31–33). Also, there is evidence to indicate gender-specific differences and similarities between men and women regarding the associations between depression, anxiety and disordered eating behaviors (34). During the COVID-19 pandemic, changes in eating attitudes have been reported by university students and also depressive symptoms were found to be associated with bulimia nervosa (35). Furthermore, reporting a gender-diverse identity as a university student was associated with depressive symptoms (36), which indicates the need to examine the prevalence and potential relation between eating attitudes and depressive symptomatology in sexual minorities during this period.

Taken together, available research supports the assumption that general psychosocial vulnerability and sexual-minority status are important for the understanding of disturbed eating attitudes and depressive symptomatology in LGBTIQ individuals. Yet, the current evidence relies mostly on research conducted in Europe and the United States and needs to be expanded to contexts with different cultural and religious backgrounds. Furthermore, most previous studies focused on the factors that increase the risk for mental health conditions in LGBTIQ individuals, and neglected the roles of potentially protective factors. Studies that take general risk and protective factors into account could provide valuable information about the unique role of sexual-minority stressors in the LGBTIQ population and enhance the theoretical conceptualizations that intend to explain mental health disparities in LGBTIQ individuals. Therefore, the current study aims to understand whether sexual-minority-related stressors explain the disturbance in eating attitudes and depressive symptomatology beyond the general psychosocial risk and protective factors in a sample of LGBTIQ individuals in Turkey.

Research demonstrated that LGBTIQ individuals perceive substantial direct and indirect discrimination in areas related to education, employment, and health care in Turkey (37). Furthermore, due to the lack of legislation related to LGBTIQ rights, most report a reluctance to pursue a legal complaint about these negative experiences and do not believe that the justice system can solve their problems (37). LGBTIQ individuals reported a significantly higher number of minority stressors that involved physical, psychological, and economical violence, and forced sexual relationships in adulthood compared to heterosexual controls in Turkey (38). They were also more likely to experience physical and sexual abuse during childhood (38). Homosexual men reported a significantly higher disturbance in eating attitudes as compared to heterosexual men (39). Identifying as homosexual or bisexual was associated with an increased risk for suicidal ideation than for heterosexual sexual identity (40). Furthermore, internalized homophobia predicted worse general health status and depressive symptomatology among the LGBTIQ individuals (41, 42).

LGBTIQ individuals experienced worse mental health regarding depression and anxiety symptoms during the COVID-19 pandemic and reported increased problem drinking behaviors in comparison to their cisgender counterparts (43). Recent studies demonstrated that pandemic-related stress aggravated eating concerns and disorders among LGBTIQ individuals (12, 44). It was found that constraints to physical exercise, challenging eating patterns, and weight concerns were related to LGBTIQ individuals' pandemic experiences. Moreover, social support, which was hindered because of the pandemic-related restrictions, was found to be protective against increased eating disorder symptoms (12). Therefore, the interpersonal relationships that already play a negative impact on these people's mental health, might have worsened during the pandemic. By evaluating eating attitudes and depressive symptoms of this Turkish sexual minority sample, which identifies as LGBTIQ, during the pandemic, this study intends to identify disorder-specific risk factors that might be useful to address and tailor mental health promotion strategies for this population.

The current study (1) investigated eating attitudes and depressive symptoms in a Turkish sexual minority who identify as LGBTIQ and (2) explored potential predictors for eating attitudes and depressive symptoms (e.g., minority stressors, appearance anxiety, appearance perfectionism, body perception, body dissatisfaction, generalized anxiety, social support, social isolation, self-efficacy, resilience). We examined whether minority stressors (e.g., heterosexist experiences, internalized homophobia) explained eating attitudes and depressive symptoms after controlling for selected psychosocial risks and protective factors. The present study provides data from a large sample of LGBTIQ individuals in Turkey and aims to disentangle the factors that heighten the risk for disturbed eating attitudes and depressive symptomatology in this population.

## Materials and methods

### Participants and procedure

Recruitment took place between February 2022 and June 2022. Multiple recruitment strategies were utilized to reach the participants of the study. LGBTIQ associations and solidarity groups in Turkey were contacted and informed about the purpose of the study. They shared the information about the research on their social media sites and accounts, and sent recruitment e-mails through their listservs. In addition, a popular online dating website for LGBTIQ individuals advertised the study. We also conducted online presentations on websites that LGBTIQ individuals frequent. Participants received a link to an online questionnaire. They had to consent to participate before they could access the questionnaires. In the consent form, participants received comprehensive information about the purpose of the study, and about the survey's anonymity and the possibility of refusing to participate. The survey took approximately 35 min to complete. The Research Ethics Committee of Bursa Uludag University approved the study.

### Measures

#### Sociodemographic characteristics

The participants were asked to indicate their age and assigned sex at birth: “Woman” (1) and “Man” (2). They responded to a question that inquired about their gender identity. The response options included: Male (1), Female (2), Trans male (3), Trans female (4), and Non-binary (e.g., diverse, genderqueer, gender nonconforming, agender, gender fluid, trans-non-binary, intersex) (5). To indicate their sexual orientation, the participants chose whether they were “Attracted to men” (1), “Attracted to women” (2), “Attracted to both men and women” (3), or “Attracted to neither men nor women” (4). They could also respond to this question with the “I don't want to respond” (5) option. Finally, we asked them to indicate whether they considered themselves part of the LGBTIQ community (“Yes” or “No”). This question was added to ensure that only the participants who identified themselves as part of the LGBTIQ community were included in the sample. Therefore, we classified participants attracted to men as “gay men” and those attracted to women as “lesbians.” Participants attracted to both men and women were classified as “bisexuals.” “Asexual” individuals were attracted to neither men nor women. Participants were also asked questions about their occupation, educational level, relationship status, whether they experience any chronic physical conditions (“Yes” or “No”), and had a history of mental disorder diagnoses (“Yes” or “No”). The participants with a history of mental disorder diagnoses were also assessed on the type of diagnoses they received. The response options involved depression, bipolar disorder, anxiety disorder, obsessive-compulsive disorder, personality disorder, eating disorder, attention deficit hyperactivity disorder, and any other mental disorder diagnoses. The participants could also respond with “I don't know” option. Multiple responses were allowed.

#### Outcome measures

##### Eating Attitudes Test-26

The Eating Attitudes Test-26 (EAT-26) (45) is the shortened version of the 40-item form of the same scale (i.e., EAT-40) (46). It is one of the most widely used scales to detect disturbances in eating patterns in both clinical and non-clinical samples. The EAT-26 consists of three parts (A, B, and C). Part A contains demographic information about the participants and their weight, height, and their lowest and highest weight; Part B contains 26 items related to eating attitudes; and Part C includes five items related to eating behaviors. The scoring for the first 25 questions that make up Part B is 3 = Always, 2 = Very often, 1 = Often, and 0 = Other answers (i.e., sometimes, rarely, never). For the last question (i.e., Question 26), reverse scoring is used. Scores of 20 and above indicate deterioration in eating patterns. Parts A and C of the scale are not included in the scoring. However, the information obtained from these sections is used to evaluate the current eating pathology. The scale consists of three factors: “diet,” “bulimia and food preoccupation,” and “oral control.”

The psychometric properties of the Turkish adaptation of the EAT-26 were examined in a sample of university students (*N* = 1,500) (47). The exploratory factor analysis revealed a three-factor structure called “Preoccupation with Eating,” “Restriction,” and “Social Pressure,” which explained 38.5% of the total variance. The confirmatory factor analysis showed that the three-factor structure was close to the acceptable fit. Significant positive correlations were found between the EAT-26 and EAT-40 and the Brief Symptom Inventory (48). In addition, the Eating Disorder Examination Questionnaire (49) and the Brief Symptom Inventory scores of those who scored above or below the cut-off score of EAT-26, differed significantly. The Cronbach's alpha internal consistency coefficient of the scale was 0.84, and the test-retest reliability coefficient was 0.78. These findings indicated that the scale could be used as a valid and reliable measurement tool to evaluate eating attitudes in Turkey. In the current study, the total scale score was used and the internal consistency was acceptable (Cronbach's α = 0.88). We also calculated the body mass index (BMI) based on the reported weight and height.

##### Patient Health Questionnaire-9

The Patient Health Questionnaire-9 (PHQ-9) is the nine-item depression module of the Patient Health Questionnaire (50). The PHQ-9 evaluates the criteria outlined in the Diagnostic and Statistical Manual of Mental Disorders-IV (DSM-IV) (51) to diagnose depression. It provides information on the frequency and severity of depressive symptoms. It is one of the most widely used scales for depressive symptoms and their severity. The response scale ranges from “0” (not at all) to “3” (almost daily). Total scores between 1 and 4 indicate normal or minimal depression, scores between 5 and 9 indicate mild depression, scores between 10 and 14 indicate moderate depression, scores between 15 and 19 indicate moderately severe depression, and scores between 20 and 27 indicate severe depression.

Turkish adaptation of the PHQ-9 was conducted on 96 patients who applied to family medicine clinics in Turkey (52). Three researchers translated the scale, and then an independent professional translator back-translated it into English. The meaning and intelligibility between the English text obtained by the back translation and the Turkish text were compared. The internal consistency coefficient calculated for the final translation of the scale was determined as 0.84. The internal consistency of the scale was acceptable in the current study (Cronbach's α = 0.91).

#### Sexual-minority stress

##### Daily Heterosexist Experiences Scale

The Daily Heterosexist Experiences Scale is a self-report measure that assesses the minority stress of lesbian, gay, bisexual, and transgender individuals (53). The scale consists of 50 items, including nine factors. Participants indicate to what extent the experiences expressed in each item disturbed or bothered them during the preceding 12 months. The items are evaluated on a 6-point Likert-type scale: 0 = Didn't happen/not applicable to me; 1 = It happened, and it bothered me not at all; 2 = It happened, and it bothered me a little bit, 3 = It happened, and it bothered me moderately; 4 = It happened and it bothered me quite a bit; 5 = It happened and it bothered me extremely. The scoring can be done in two ways. First, after re-coding the answers as 0 = 0 and all other answers (i.e., 1, 2, 3, 4, 5) = 1, a total score is obtained to determine how many heterosexist experiences have occurred. After re-coding the answers with 0, 1 = 0, and all the other answers remaining the same, the results show the average level of distress experienced by the participants in the face of heterosexist experiences. The nine factors of the scale were “Gender Expression,” “Vigilance,” “Parenting,” “Discrimination/Harassment,” “Vicarious Trauma,” “Family of Origin,” “HIV/AIDS,” “Victimization,” and “Isolation.” The Cronbach's alpha internal consistency coefficient of the total scale score was calculated as 0.92. The Cronbach's alpha internal consistency coefficients calculated for the subscales ranged between 0.76 and 0.87. The score obtained from the scale was moderately associated with psycho-social distress (i.e., depression, anxiety, post-traumatic stress disorder, perceived stress level).

Since the adaption of the scale into Turkish has not yet taken place, we translated and back-translated it before administering it in the current study. Most LGBT individuals who are parents keep their gender identities and sexual orientation secret in Turkey. Therefore, we excluded the six items under the “Parenting” factor that assess the perceived anti-LGBT discrimination related to LGBT individuals' children and parenting. The scale developers also recommended determining the subscales to administer based on the study's objectives (53). We calculated both the number of and the distress related to daily heterosexist experiences in the current study. The internal consistency for the number of daily heterosexist experiences was 0.97. The internal consistency for the distress related to heterosexist experiences was 0.96.

##### Internalized Homophobia Scale

The Internalized Homophobia Scale is a nine-item scale developed to determine the internalization of homophobic attitudes among gay men (6). Later, separate forms for lesbian women and bisexual women and men were developed (54–56). Each item is scored on a 5-point Likert-type scale (1 = strongly disagree, 5 = strongly agree). Higher scores indicate increased internalized sexual stigma and negative attitudes toward the self. Individuals with higher scores had low self-esteem and less openness to heterosexual people about their sexual orientation. They were less satisfied with homosexual friends and communities and more likely to associate personal failures with homophobic prejudices (57). The Turkish adaptation of the scale was carried out on a sample of 112 gay men and 20 bisexual male university students (58). The internal consistency (0.82) and split-half reliability (0.82) of the scale were good, and it had a single-factor structure similar to the original study. The scores obtained from the scale were associated with psychological problems, especially depression and anxiety symptoms. In addition, the scale scores were positively related to negative affect and negatively associated with self-esteem.

In the current study, participants who were “attracted to men” and “attracted to women” received the forms for gay men and lesbian women, respectively. Those who indicated their sexual orientation as “attracted to both men and women” received bisexual forms of the questionnaire, depending on their reported gender identity. Participants who identified as “female” received the bisexual female form. In contrast, participants who identified as “male” received the bisexual male form. Individuals who identified their gender identity as non-binary and responded to the sexual-orientation question as “attracted to both men and women” received the scale based on their sex at birth. Participants whose assigned sex was “woman” received the bisexual female form, whereas participants whose assigned sex was “man” received the bisexual male form. Participants who responded to the sexual-orientation question with “attracted to neither men nor women” and “I don't want to respond” did not receive the questionnaire. The scale's internal consistency was acceptable for gay men (0.88), lesbian women (0.75), bisexual female (0.89), and bisexual male (0.84) forms. A composite score was used as the measure for internalized homophobia in the current study.

#### Potential predictors of eating attitudes and depressive symptomatology

##### Social Appearance Anxiety Scale

The Social Appearance Anxiety Scale is a self-report measure that assesses the cognitive, emotional, and behavioral anxiety related to social appearance (59). The scale consists of 16 items that are evaluated on a 5-point Likert scale (1 = never; 5 = extremely). Higher scores indicate increased anxiety about social appearance. Higher scores were also found to be associated with the fears of negative evaluation and depression. The Turkish adaptation of the scale was conducted with 340 university students (60). The scale had a single-factor structure similar to its original form. The Cronbach's alpha internal consistency coefficient was 0.93, the test-retest reliability coefficient was 0.85, and the split-half reliability coefficient was 0.88. In the current sample, the scale had acceptable internal consistency: 0.95.

##### Physical Appearance Perfectionism Scale

The Physical Appearance Perfectionism Scale is a self-report scale developed to capture both the positive and negative aspects of physical appearance perfectionism (61). It consists of 12 items that are evaluated on a 5-point Likert scale (1 = strongly disagree; 5 = strongly agree). As the score obtained from the scale increases, perfectionism about physical appearance increases. The exploratory and confirmatory factor analyses indicated a two-factor structure for the scale. The two factors were “Worry About Imperfection” and “Hope for Perfection.” The “Worry About Imperfection” factor was associated with a negative-appearance evaluation and concerns about body image and weight. The “Hope for Perfection” factor, on the other hand, was positively associated with the striving dimension of perfectionism and better self-image. The Turkish adaptation of the scale was conducted on 320 volunteers (62). The findings showed that the scale had a two-factor structure similar to its original form. Cronbach's alpha coefficients were 0.90 and 0.93 for the “Worry About Imperfection” and “Hope for Perfection” factors. In the current sample, the scale had acceptable internal consistency: 0.89.

##### Figure Rating Scale

The Figure Rating Scale consists of 18 schematic silhouettes (nine women and nine men) that range from very thin to obese (63). The scale is widely used to measure body perception and dissatisfaction. The respondents indicate which of the nine silhouettes best reflects their own body. Then they are asked to choose the figure that reflects their ideal body size. The discrepancy between the figure that reflects the respondents' own body (i.e., perceived body mass index) and the figure that reflects their ideal body (i.e., ideal body mass index) provides the measurement for body dissatisfaction. In addition, the body perception index is calculated by multiplying the ratio of the body mass index perceived to the actual body mass index by 100. The body perception index determines whether the individual evaluates their perceived body mass index realistically. The test-retest reliabilities for the perceived body mass index and ideal body mass index ranged between 0.81–0.92 and 0.71–0.82, respectively (64). In the current study, the participants were presented with women's and men's silhouettes and instructed to indicate their ideal and perceived body mass index based on the gender with which they identified. We calculated body dissatisfaction based on the discrepancy between perceived and ideal body mass index.

##### Generalized Anxiety Disorder-7

The Generalized Anxiety Disorder-7 (GAD-7) is a short self-report test that evaluates generalized anxiety disorder criteria according to the DSM-IV classification (65). The scale consists of seven items that inquire about common anxiety symptoms experienced in the preceding 2 weeks. The items are evaluated on a 4-point Likert-type scale (0 = never, 1 = some days, 2 = more than half of the days, 3 = almost every day). Total scores of 5, 10, and 15 are determined to be the cut-off points for mild, moderate, and severe anxiety, respectively. Higher scores were strongly associated with multiple domains of functional impairment (i.e., general health scales and disability days). Although generalized anxiety disorder and depression symptoms are often together, it was shown that generalized anxiety disorder and depression symptoms had independent effects on functional impairment and disability. The Turkish adaptation of the GAD-7 was conducted with 110 patients who had been diagnosed with generalized anxiety and 112 healthy control group participants (66). The findings indicated that the scale had a single factor structure similar to the original test. The most acceptable cut-off value for the GAD-7 test was found to be 8. The Cronbach's alpha internal consistency coefficient for the total scale score was calculated as 0.85. In the current sample, the scale had acceptable internal consistency: 0.94.

##### ENRICHD Social Support Inventory

The ENRICHD Social Support Inventory is a self-report measure developed within the Enhancing Recovery in Coronary Heart Disease (ENRICHD) project (67). It determines the amount of social support in the lives of patients with coronary heart disease. The scale consists of seven items and it is evaluated on a 5-point Likert scale (1 = never, 5 = always). The “Yes” answer to the last question (i.e., “Are you married or living with a partner?”) is calculated as 4 points, and the “No” answer as 2 points. The higher the score on the scale, the higher the perceived social support. The scores obtained from the scale were correlated with other scales that measure perceived emotional support. Since it is a short scale with good psychometric properties, it is often used for screening purposes to measure the amount of perceived emotional and functional social support in different samples (68). A Turkish adaption of the scale has not been conducted. Therefore, we translated and back-translated it for the current study. The internal consistency of the scale was acceptable: 0.90.

##### UCLA Three-item Loneliness Scale

The UCLA Three-item Loneliness Scale (69) was developed from the Revised UCLA Loneliness Scale (70). This shortened screening tool demonstrated similar psychometric properties to the 20-item Revised UCLA Loneliness Scale. It addresses the lack of friendship, feeling excluded, and feelings of isolation from others. Each item is rated on a 3-point Likert-type scale (1 = Almost never, 2 = Sometimes, 3 = Often). All items are summed to give the total score. The Three-item Loneliness Scale provides a quick and concise method to collect information about social isolation. Total scores range from 3 to 9, and scores above 6 indicate that individuals feel lonely. A Turkish adaption of the scale has not been conducted. Therefore, we translated and back-translated it for the current study. The internal consistency of the scale was acceptable: 0.86.

##### General Self-efficacy Scale

The General Self-efficacy Scale is designed to assess the positive self-beliefs that capture an individual's capacity to cope with various challenges and demands in life (71). It has 10 items that are evaluated on a 4-point Likert scale (1 = Totally false, 4 = Totally true), where higher scores indicate increased self-efficacy beliefs. The scale's psychometric properties were examined in 25 countries and showed a single-factor structure (72). The Turkish adaptation of the scale was conducted with 693 university students aged 17–39 (73). The Turkish version of the scale was found to have a two-factor structure with “Effort and Resistance” and “Ability and Confidence” categories. The total scale score's internal consistency and test-retest reliability coefficients were calculated as 0.83 and 0.80, respectively. The internal consistency of the scale was acceptable in the current sample: 0.93.

##### Brief Resilience Scale

The Brief Resilience Inventory is a six-item scale developed to measure the ability to overcome stress and self-recovery (74). Each item is rated on a 5-point Likert scale (1 = strongly disagree, 5 = strongly agree). The total score varies between 6 and 30. Higher scores indicate psychological resilience. The scores obtained from the scale indicated a single factor structure and they were negatively related to anxiety, depression, negative emotions, and perceived stress. The Turkish adaptation of the scale was conducted on a university sample with acceptable fit indices for the single-factor structure similar to the original study (75). The internal consistency of the scale was low in the current study: 0.14.

### Statistical analysis

Descriptive statistics were run for the sociodemographic characteristics on the whole sample and the sample of completers. Participants were considered completers if they provided data on at least one of the outcome measures (i.e., PHQ-9 or EAT-26). Subgroup analyses were conducted with chi-square-tests or Fisher's exact test (when more than 20% of the cells had expected frequencies < 5) to identify differences between the completers and the drop-outs regarding sociodemographic characteristics. Subgroup analyses were also conducted on the sample of completers regarding the differences in sociodemographic characteristics by sexual orientation (i.e., lesbian, gay, bisexual, asexual). The Standardized Pearson Residuals were used to decompose the effect of significant test statistics (76). To gauge the effect size, the ϕ-coefficient was calculated, while Cramér's V (ϕ_c_) was used when the contingency table was larger than 2 × 2, with ϕ, ϕ_c_ =0.10 indicating a small effect, ϕ, ϕ_c_ =0.30 an average effect, and ϕ, ϕ_c_ =0.50 a large effect (77).

A three-stage multiple hierarchical regression analysis was conducted with eating attitudes (EAT-26) as the dependent variable. Age, assigned sex at birth, and sexual orientation were entered in the first step to control for sociodemographic characteristics. To examine the unique contribution of minority stress on eating attitudes, variables related to depression (i.e., Patient Health Questionnaire-9), anxiety symptoms (i.e., Generalized Anxiety Disorder-7), body image (i.e., Figure Rating Scale, Social Appearance Anxiety Scale, Physical Appearance Perfectionism Scale), social support (i.e., ENRICHD Social Support Inventory), social isolation (i.e., UCLA Three-item Loneliness Scale), self-efficacy (i.e., General Self-efficacy Scale), and resilience (i.e., Brief Resilience Scale) were entered in the second step. In the third step, both the number of and the distress related to heterosexist experiences from the Daily Heterosexist Experiences Scale and the scores of the Internalized Homophobia Scale were entered. The same procedure was followed to predict depressive symptomatology. The sociodemographic characteristics were entered in the first step, disorder-specific risk factors were entered in the second step, and variables related to sexual minority stress were entered in the final step.

Prior to the analyses, collinearity and multivariate outliers were examined. The collinearity statistics revealed that tolerance and variance inflation factor (VIF) statistics were within the acceptable limits for the independent variables (tolerance values were above 0.2 and VIF values were <4). An examination of the Mahalanobis distance scores indicated no multivariate outliers and the inspection of residual and scatter plots for both dependent variables confirmed that the normality, linearity, and homoscedascity assumptions were met. The analyses were run using the Statistical Package for Social Sciences (SPSS) version 28 (78). A two-tailed α = 0.05 was applied to statistical testing.

## Results

### Sociodemographic characteristics

Overall, 477 participants started the online questionnaire. Thirty-seven participants gave online consent to participate but did not provide further data. Thus, the total sample size consisted of 440 participants. The mean age of the participants was 31.92 (*SD* = 11.82). The majority of the participants (64.3%) reported that their current gender identity was male. This was followed by non-binary (14.1%), female (13.9%), trans female (4.1%), and trans male (3.6%) gender identities. For 79.7% of the participants, assigned sex at birth was man (*n* = 439). The data on sexual orientation were available for 433 participants. Of these, 62.8% (*n* = 272) reported being attracted to men, 23.6% (*n* = 102) were attracted to both men and women, 7.2% (*n* = 31) were attracted to women, and 3% (*n* = 13) were attracted to neither men nor women. Fifteen participants (3.5%) did not want to respond to this question. More than half of the participants were single (58.9%, *n* = 259). For the remaining, 111 (25.2%) reported being in a relationship, 27 (6.1%) were divorced, 27 (6.1%) were widowed, and 16 (3.1%) were married. The information on occupational and educational status were provided by 434 and 426 participants, respectively. The majority of the sample were workers and civil servants (37.6%), students (24.4%), and unemployed (17.3%). The percentage of participants who had at least a high school education was 86.9, indicating reasonably high educational status. The majority of the participants had not received a mental disorder diagnosis in the past (87.6%; total *n* = 434) and they were not suffering from a chronic physical condition (86.1%; total *n* = 433). Of the received mental disorder diagnoses, depression was the most frequently reported mental disorder diagnoses (57.4%). This was followed by anxiety disorder (31.5%), bipolar disorder (27.4%), obsessive-compulsive disorder (20.4%), attention deficit hyperactivity disorder (20.4%), personality disorder (7.4%) and eating disorder (7.4%). Also, 7.4% mentioned that they had received another mental disorder diagnoses and 3.5% responded with “I don't know” option.

Of the 440 participants, *N* = 237 participants provided data on at least one of the outcome measures (i.e., PHQ-9 or EAT-26) and they were considered completers. There were significant differences between the completers (*N* = 237) and non-completers (*N* = 240) regarding age (t (437) = 2.206, *p* = 0.028), gender identity [χ^2^(4) = 46.554, *p* < 0.001; ϕ_c_ =0.325], assigned sex at birth [χ^2^(1) = 38.493, *p* < 0.001; ϕ =0.296], and sexual orientation [χ^2^(4) = 52.558, *p* < 0.001; ϕ_c_ =0.348]. The completers were younger and more likely to report their gender identity as male and their assigned sex at birth as man. They were also more likely to report their sexual orientation as gay. On the other hand, female, trans male, trans female, and non-binary gender identities were more frequently mentioned by the non-completers. Their assigned sex at birth was more likely woman and they more often reported their sexual orientation as lesbian, bisexual, and asexual. The groups also differed in their educational level [χ^2^(2) = 31.713, *p* < 0.001; ϕ_c_ = 0.273], relationship status [χ^2^(4) = 18.204, *p* = 0.001, ϕ_c_ = 0.203], history of mental disorder [χ^2^(1) = 13.357, *p* < 0.001; ϕ = −0.175], and the presence of a chronic physical condition [χ^2^(1) = 5.199, *p* = 0.025; ϕ = −0.110]. No education and less than a high school education was more frequently reported by non-completers. In contrast, completers more frequently reported at least high school and higher educational degrees. Being single was more commonly reported among the completers, whereas widows were more common among the non-completers. Finally, the completers were more likely to have received a mental disorder diagnosis in the past and to suffer from a chronic physical condition.

The remaining analyses were conducted on the completers (*N* = 237). Most reported their sexual orientation as attracted to men (*N* = 181). It was followed by those attracted to men and women (*N* = 43) and those attracted to only women (*N* = 11). None of the completers reported being asexual. Two participants did not want to answer the question about their sexual orientation. Table 1 represents the sociodemographic characteristics of the completers and the differences by sexual orientation.

**Table 1:** Sociodemographic characteristics of the whole sample and differences by sexual orientation (*N* = 237).

	**Completers**	**Attracted to men**	**Attracted to women**	**Attracted to both**	***p***
	**(*N* = 237)**	**(*N* = 181)**	**(*N* = 11)**	**(*N* = 43)**	
	**Mean (*SD*)**	**Mean (*SD*)**	**Mean (*SD*)**	**Mean (*SD*)**	
Age	30.77 (9.31)	31.04 (8.53)	29.55 (14.89)	30.37 (10.86)	0.819
Gender identity					**<0.001**
Female	8.4%	3.3%	63.6%	14%	
Male	78.5%	84%	18.2%	74.4%	
Trans male	1.7%	0.6%	9.1%	4.7%	
Trans female	1.7%	2.2%	0%	0%	
Non-binary	9.7%	9.9%	9.1%	7%	
Assigned sex at birth					**<0.001**
Woman	9.3%	2.8%	63.6%	20.9%	
Man	90.7%	97.2%	36.4%	79.1%	
BMI	24.65 (4.38)	24.60 (4.32)	23.21 (5.64)	24.98 (4.37)	0.553
Relationship status					**<0.001**
Married	4.6%	5%	9.1%	2.3%	
In relationship	21.9%	17.1%	54.5%	34.9%	
Widowed	3%	2.2%	18.2%	2.3%	
Single	65.8%	71.3%	9.1%	55.8%	
Divorced	4.6%	4.4%	9.1%	4.7%	
Occupation					0.728
Student	21.9%	20.4%	27.3%	27.9%	
Unemployed	16.9%	17.7%	18.2%	11.6%	
Worker/Civil servant	36.7%	38.1%	27.3%	32.6%	
Self-employed	21.1%	19.9%	27.3%	25.6%	
Housewife	0.8%	0.6%	0%	2.3%	
Retired	2.5%	3.3%	0%	0%	
Education					**0.002**
No education/Primary school	1.7%	0.6%	18.2%	2.4%	
Middle/High school	3%	30.7%	36.4%	50%	
University or higher	95.2%	68.8%	72.7%	88.1%	
History of mental disorder	17.7% (Yes)	18.8% (Yes)	18.2% (Yes)	11.6% (Yes)	0.565
Chronic physical condition	17.3% (Yes)	14.4% (Yes)	45.5% (Yes)	18.6% (Yes)	**0.030**

BMI, body mass index. History of mental disorder diagnosis and chronic physical condition were assessed with “Yes”/”No” answers. Significant p values are shown in boldface, *p* < 0.05.

The Fisher's exact test statistics indicated that the participants' gender identity (*p* < 0.001; ϕ_c_ = 0.367) and assigned sex at birth (*p* < 0.001; ϕ_c_ = 0.490) differed significantly by sexual orientation. For the participants attracted to men, the assigned sex at birth was more likely to be man. They were also more likely to report their gender identity as male. By comparison, participants attracted to women more frequently had their assigned sex at birth as woman. They were also more likely to identify as female than as male. Thus, the participants attracted to men and those attracted to women more frequently indicated their gender identities aligned with their assigned sex at birth. Participants attracted to both men and women were more likely to report their assigned sex at birth as women than participants who were attracted to men. Overall, four participants reported their gender identity as trans female, and all indicated their sexual orientation as attracted to men. Another four identified as trans males. Of these, two reported their sexual orientation as attracted to both men and women, one as attracted to men, and one to women.

There were significant differences between the groups regarding their relationship (*p* < 0.001; ϕ_c_ = 0.243) and educational (*p* = 0.002; ϕ_c_ = 0.233) status. Participants attracted to men were more likely to be single and less likely to be in a relationship than both participants attracted to women and participants attracted to both men and women. They were also more likely to report university or higher academic degrees. Lastly, the presence of chronic physical conditions differed significantly by sexual orientation (*p* = 0.030; ϕ = 177). Participants who were attracted to women suffered significantly more from chronic physical conditions.

### Predictors of eating attitudes

The hierarchical multiple regression analysis to predict eating attitudes at Stage 1 revealed that sociodemographic characteristics contributed significantly to the model [R^2^ = 0.18, F_(4,174)_ = 9.88, *p* < 0.001]. Introducing variables related to depression, anxiety, body image, social support, social isolation, self-efficacy, and resilience was associated with a significant additional 13% variation in Stage 2 [F_(9,165)_ = 3.41, *p* = 0.001]. The model was also statistically significant [R^2^ = 0.31, F_(13,165)_ = 5.78, *p* < 0.001)]. In Stage 3, variables related to sexual-minority stress accounted for a significant additional 12% variation in eating attitudes [F_(3,162)_ = 11.24, *p* < 0.001]. The final model was statistically significant [R^2^ = 0.43, F_(16,162)_ = 7.68, *p* < 0.001]. The summary of the findings for the prediction of eating attitudes is shown in Table 2.

**Table 2:** Summary of the multiple hierarchical regression analysis predicting eating attitudes (*N* = 237).

**Variables**	***b***	**95% CI^a^**	**β**	**t**	***p***
**Block 1**					
Age	−0.04	−0.21; 0.13	−0.03	−0.47	0.638
Assigned sex at birth	−10.85	−18.18; −3.52	−0.24	−2.92	**0.004**
Sexual orientation					
Attracted to women vs. men	13.52	3.91; 23.13	0.23	2.78	**0.006**
Attracted to both vs. men	3.46	−0.98; 7.89	0.11	1.54	0.126
**Block 2**					
Age	0.14	−0.04; 0.33	0.113	1.56	0.121
Assigned sex at birth	−6.64	−13.96; 0.69	−0.15	−1.79	0.075
Sexual orientation					
Attracted to women vs. men	15.91	6.38; 25.44	0.27	3.3	**0.001**
Attracted to both vs. men	2.68	−1.55; 6.92	0.09	1.25	0.213
Depressive symptomatology (PHQ-9)	0.32	−0.07; 0.71	0.18	1.61	0.108
Anxiety (GAD-7)	−0.14	−0.6; 0.32	−0.07	−0.60	0.548
Social appearance anxiety (SAAS)	0.07	−0.1; 0.24	0.09	0.85	0.398
Physical appearance perfectionism (PPS)	0.01	−0.20; 0.23	0.01	0.13	0.895
Body dissatisfaction (FRS)	0.33	−0.59; 1.26	0.05	0.71	0.476
Social Support (ENRCHD)	0.00	−0.24; 0.24	0.00	0.03	0.977
Self-efficacy (GSE)	0.16	−0.09; 0.42	0.1	1.26	0.211
Resilience (BRS)	−0.31	−0.85; 0.24	−0.08	−1.11	0.267
Social isolation (UCLA)	1.48	0.32; 2.63	0.26	2.52	**0.013**
**Block 3**					
Age	0.10	−0.06; 0.27	0.08	1.22	0.223
Assigned sex at birth	−9.18	−15.99; −2.37	−0.20	−2.66	**0.009**
Sexual orientation					
Attracted to women vs. men	4.69	−4.86; 14.24	0.08	0.97	0.333
Attracted to both vs. men	0.35	−3.72; 4.42	0.01	0.17	0.864
Depressive symptomatology (PHQ-9)	0.38	0.01; 0.74	0.22	2.04	**0.043**
Anxiety (GAD-7)	−0.14	−0.56; 0.28	−0.07	−0.66	0.508
Social appearance anxiety (SAAS)	0.06	−0.1; 0.23	0.08	0.8	0.424
Physical appearance perfectionism (PPS)	0.05	−0.15; 0.25	0.04	0.46	0.644
Body dissatisfaction (FRS)	0.57	−0.29; 1.42	0.08	1.3	0.196
Social support (ENRCHD)	−0.03	−0.25; 0.19	−0.02	−0.26	0.791
Self-efficacy (GSE)	0.23	−0.02; 0.47	0.14	1.83	0.069
Resilience (BRS)	−0.35	−0.85; 0.15	−0.09	−1.37	0.171
Social Isolation (UCLA)	1.38	0.31; 2.45	0.24	2.55	**0.012**
Number of heterosexist experiences (DHES)	0.73	0.47; 0.99	0.57	5.56	**<0.001**
Distress related to heterosexist experiences (DHES)	−0.14	−0.20; −0.09	−0.52	−4.98	**<0.001**
Internalized homophobia (IHS)	0.04	−0.11; 0.2	0.03	0.54	0.588

PHQ-9, Patient Health Questionnaire-9; GAD-7, Generalized Anxiety Disorder-7; SAAS, Social Appearance Anxiety Scale; PPS, Physical Appearance Perfectionism Scale; ENRCHD, ENRICHD Social Support Inventory; GSE, General Self-Efficacy Scale; BRS, Brief Resilience Scale; UCLA, UCLA 3-item Loneliness Scale; DHES, Daily Heterosexist Experiences Scale; IHS, Internalized Homophobia Scale.

Significant *p* values are shown in boldface.

a 95% confidence interval for b.

The results demonstrated that participants whose assigned sex at birth was woman and participants who were attracted to men were more likely to report deterioration in their eating attitudes in Stage 1 of the analyses. In Stage 2, only sexual orientation and social isolation were significant predictors of eating attitudes. Participants who were attracted to men and participants who reported increased social isolation were more likely to experience deterioration in their eating attitudes. When all of the variables were entered in the final stage, disturbance in eating attitudes was predicted by assigned sex at birth, depression, social isolation, and daily heterosexist experiences. Participants whose assigned sex at birth was woman and who reported higher depression, social isolation, and daily heterosexist experiences and lower distress related to heterosexist experiences were more likely to report disturbance in eating attitudes.

### Predictors of depressive symptomatology

The hierarchical multiple regression analysis to predict depressive symptomatology at Stage 1 revealed that sociodemographic characteristics contributed significantly to the model [R^2^ = 0.19, F_(4,174)_ = 10, *p* < 0.001)]. Introducing variables related to eating attitudes, anxiety, body image, social support, social isolation, self-efficacy, and resilience was associated with a significant additional 49% variation in Stage 2 [F_(4,165)_ = 28,51, *p* < 0.001]. The model was statistically significant [R^2^ = 0.68, F_(13,165)_ = 27.2, *p* < 0.001]. In the final stage, variables related to sexual-minority stress accounted for an additional 2% variation. The final model was statistically significant [R^2^ = 0.70, F_(16,162)_ = 23.42, *p* < 0.001] and indicated that the inclusion of variables related to sexual-minority stress improved the model prediction [F_(3,162)_ = 2.93, *p* = 0.035]. The summary of the findings for the prediction of symptoms is represented in Table 3.

**Table 3:** Summary of the multiple hierarchical regression analysis predicting depressive symptomatology (*N* = 237).

**Variables**	***b***	**95% CI^a^**	**β**	**t**	***p***>
**Block** 1					
Age	−0.26	−0.36; −0.16	−0.36	−5.21	**<0.001**
Assigned sex at birth	−6.61	−10.81; −2.41	−0.26	−3.11	**0.002**
Sexual orientation					
Attracted to women vs. men	−4.37	−9.88; 1.13	−0.13	−1.57	0.119
Attracted to both vs. men	1.05	−1.49; 3.59	0.06	0.81	0.417
**Block 2**					
Age	−0.06	−0.12; 0.01	−0.08	−1.58	0.117
Assigned sex at birth	−3.86	−6.69; −1.03	−0.15	−2.7	**0.008**
Sexual orientation					
Attracted to women vs. men	−5.73	−9.47; −1.99	−0.17	−3.03	**0.003**
Attracted to both vs. men	0.33	−1.33; 1.99	0.02	0.39	0.695
Eating attitudes (EAT-26)	0.05	−0.01; 0.11	0.08	1.61	0.108
Anxiety (GAD-7)	0.73	0.59; 0.87	0.61	10.21	**<0.001**
Social appearance anxiety (SAAS)	0.03	−0.03; 0.10	0.08	1	0.317
Physical appearance perfectionism (PPS)	0.09	0.01; 0.17	0.13	2.15	**0.033**
Body dissatisfaction (FRS)	−0.22	−0.58; 0.14	−0.05	−1.19	0.234
Social Support (ENRCHD)	−0.08	−0.17; 0.01	−0.09	−1.74	0.083
Self-efficacy (GSE)	−0.01	−0.12; 0.09	−0.02	−0.3	0.765
Resilience (BRS)	−0.12	−0.34; 0.09	−0.06	−1.15	0.252
Social Isolation (UCLA)	−0.14	−0.60; 0.31	−0.04	−0.62	0.533
**Block 3**					
Age	−0.06	−0.13; 0.01	−0.08	−1.62	0.107
Assigned sex at birth	−3.50	−6.36; −0.65	−0.14	−2.42	**0.017**
Sexual orientation					
Attracted to women vs. men	−4.57	−8.51; −0.63	−0.13	−2.29	**0.023**
Attracted to both vs. men	0.09	−1.61; 1.8	0.00	0.11	0.913
Eating attitudes (EAT-26)	0.07	0.00; 0.13	0.11	2.04	**0.043**
Anxiety (GAD-7)	0.69	0.55; 0.83	0.58	9.72	**<0.001**
Social appearance anxiety (SAAS)	0.02	−0.04; 0.09	0.05	0.71	0.480
Physical appearance perfectionism (PPS)	0.07	−0.01; 0.16	0.11	1.78	0.076
Body dissatisfaction (FRS)	−0.18	−0.54; 0.18	−0.04	−0.99	0.325
Social Support (ENRCHD)	−0.08	−0.17; 0.01	−0.08	−1.67	0.097
Self-efficacy (GSE)	−0.00	−0.11; 0.1	−0.00	−0.07	0.943
Resilience (BRS)	−0.12	−0.33; 0.09	−0.06	−1.16	0.249
Social Isolation (UCLA)	−0.18	−0.64; 0.27	−0.06	−0.8	0.428
Number of heterosexist experiences (DHES)	−0.08	−0.20; 0.03	−0.11	−1.39	0.167
Distress related to heterosexist experiences (DHES)	0.03	0.00; 0.05	0.18	2.24	**0.027**
Internalized homophobia (IHS)	0.06	−0.00; 0.13	0.09	1.95	0.053

EAT-26, Eating Attitudes Test-26; GAD-7, Generalized Anxiety Disorder-7; SAAS, Social Appearance Anxiety Scale; PPS, Physical Appearance Perfectionism Scale; ENRCHD, ENRICHD Social Support Inventory; GSE, General Self-efficacy Scale; BRS, Brief Resilience Scale; UCLA, UCLA 3-item Loneliness Scale; DHES, Daily Heterosexist Experiences Scale; IHS, Internalized Homophobia Scale. Significant *p* values are shown in boldface.

a 95% confidence interval for b.

The findings demonstrated that assigned female sex at birth was associated with depressive symptoms at all stages of the analyses. Although younger age was associated with depressive symptomatology at Stage 1, it was not a significant predictor after the inclusion of variables at the second and third steps of the analyses. In Stage 2, higher generalized anxiety and physical appearance perfectionism predicted depression symptoms. Also, participants attracted to women were more likely to experience depressive symptoms. When all of the variables were entered in the final stage, depression symptoms were predicted by assigned sex at birth, sexual orientation, eating attitudes, generalized anxiety, and distress related to daily heterosexist experiences. Participants whose assigned sex at birth was woman and who reported being attracted to women were more likely to experience depressive symptoms. Higher disturbance in eating attitudes and increased generalized anxiety was associated with depressive symptomatology. In addition, participants who experienced higher distress related to daily heterosexist experiences were more likely to report depression symptoms.

## Discussion

This study presents the first comprehensive information about eating attitudes and depressive symptoms during the COVID-19 pandemic in a Turkish LGBTIQ sample.

A heterogeneous and large sample was recruited *via* community networks in Turkey and the participants filled out an online survey. There were differences between the completers and participants who dropped-out and did not finish the online survey. Completers were mostly younger, had male as their gender identity, assigned sex as man at birth, were attracted to men, and single. Considering that none of the completers reported being asexual, the recruitment procedures, which included ads on a Turkish dating website, could have been flawed. In addition, completers had higher education levels, they were more likely to have received a mental disorder diagnosis in the past, and more likely to suffer from a chronic physical condition, which could indicate that the participants who completed the survey probably tend to value the importance of this research and research topic. To sum up, there were differences between the completers and the people who did not complete the survey. Nevertheless, a fairly large and heterogeneous sample was recruited in the Turkish LGBTIQ community.

### Predictors of eating attitudes

Participants who reported female sex at birth and higher scores for depression and social isolation were more likely to experience a disturbance in their eating attitudes. There was a significant association between the number of heterosexist experiences and eating attitudes. Participants who reported a higher number of heterosexist experiences were more likely to report deterioration in their eating attitudes. By comparison, the distress related to heterosexist experiences was negatively associated with eating attitudes. Participants with higher distress related to heterosexist experiences were less likely to report a disturbance in their eating attitudes.

Although there were more participants assigned at birth as man, in line with previous findings, this study highlighted the female sex at birth and its proneness to eating disorders (9, 14). The intersection among the psychosocial experiences of eating disorders in populations with identity-related experiences, such as harassment, heterosexism, and internalized minority stress, as posed by the “tripartite influence model” (17), could clarify the significant association found between the number of heterosexist experiences and the deterioration in eating attitudes. Furthermore, the findings that showed social isolation and depressive symptoms as significant predictors of disturbance in eating attitudes align with the interpersonal theory of eating disorders (15), which highlights the inadequacy of social interactions that lead to negative affect to trigger or maintain eating disorders. Previous studies found that isolation and depression are significant risk factors for disordered eating behaviors in LGBTIQ individuals (5). Our findings also align with a previous study which showed that social support which was hindered because of the pandemic-related restrictions, was protective against increased eating disorder symptoms among the LGBTQ+ (12).

On the other hand, it was surprising that participants with higher distress related to heterosexist experiences were less likely to report disturbances in their eating attitudes. In the current study, the data were collected through LGBTIQ associations, solidarity groups, and a popular online dating website for LGBTIQ individuals. The participants were highly educated and most probably more accepting of their sexual-minority status. The distress related to heterosexist experiences was revealed by how bothered participants felt when faced with these experiences. It might be possible that the acknowledgment of negative feelings about heterosexist experiences in our highly educated sample was protective against disordered eating attitudes as it might have facilitated the utilization of effective coping strategies. A previous study showed coping *via* internalization as a significant intrapsychic risk factor for disordered eating behaviors (79).

Overall, current findings are in line with previous research regarding the significant role of depressive symptoms and social belongingness in eating attitudes among the LGBTIQ (9, 12) and highlights the vulnerability of female sex at birth to eating disorders even amidst a sample mostly comprised by male sex in our sample of LGBTIQ individuals. It also highlights that the number of heterosexist experiences is crucial in understanding maladaptive eating attitudes beyond the psychosocial risk and protective factors in this population.

### Predictors of depressive symptomatology

Results indicated that previously reported risk factors in heterosexual samples, such as female sex (80), generalized anxiety (81), and disturbed eating attitudes (82), were significant predictors of depressive symptomatology in this Turkish LGBTIQ sample. As compared to being attracted to men, participants who were attracted to women were more likely to report depression symptoms. In addition, higher distress related to daily heterosexist experiences was associated with depressive symptomatology, as would be expected for people who internalize prejudicial attitudes and, thus, present self-stigma as posed by the minority stress theory (6).

A previous study that stratified sexual orientation by sex reported higher rates of depression for bisexual females (29). Our findings supported female assigned sex but not bisexual orientation, as a significant predictor for depression symptoms. The comorbidity between eating disturbance and depressive symptomology and the higher prevalence of both conditions among women is attributed to the cultural ideal of thinness (83). Thin idealization occurs more frequently among women and sets the stage for increased body dissatisfaction and depressive symptomatology. In turn, disordered eating behaviors are viewed as ineffective coping strategies for depressed women in response to unattainable beauty ideals. Our findings showed disturbed eating attitudes, assigned female sex and lesbian sexual orientation as risk factors for depressive symptomatology in our sample of LGBTIQ individuals. These findings might suggest that thin idealization is a mechanism to explain why the increased depression symptoms and eating disturbances in heterosexual women might apply similarly to LGBTIQ individuals of female sex and lesbian sexual orientation.

Weight bias is a crucial concept to understand negative emotions and judgmental evaluations relating to one's body image, shape and weight. It refers to the negative weight-related evaluations of overweight and obese individuals (84). Previous research demonstrated an association between sexual minority status and the internalization of weight biases (85, 86). In a recent study, the connections between weight bias, eating concerns and depression symptoms were more pronounced in sexual minority individuals as compared to their cisgender counterparts (87). Furthermore, bisexual and lesbian women reported worst psychological wellbeing concerning eating attitudes and depressive symptoms (87). These previous findings highlight that weight bias could be an important factor in understanding eating attitudes and depressive symptoms in LGBTIQ individuals. Also, our findings might stimulate further research to examine whether LGBTIQ individuals of female sex and lesbian sexual orientation could be more prone to internalize weight biases.

Similar to the findings reported for heterosexual adults (81), generalized anxiety was a risk factor for depressive symptoms among LGBTIQ individuals in the current study. In a qualitative study, LGBT individuals with self-injurious and suicidal behaviors stated increased tension between the way that they learned how to present themselves and the impression they made on others (88). Considering the issues of discrimination toward the LGBTIQ community in Turkey (37, 89), we believe that a proportion of the increase in the generalized anxiety scores might be attributed to the minority stressors in the current sample. This interpretation is supported by the finding that demonstrated that higher distress related to heterosexist experiences was predictive of depressive symptomatology beyond the psychosocial risk factors. It is possible that, for LGBTIQ individuals who are prone to developing depression symptoms, managing the distress related to daily heterosexist experiences was counteracted by ineffective emotional coping strategies. These findings might point to the need for interventions in the mental field that empowers LGBTIQ individuals' skills in coping with unpleasant emotions related to heterosexist experiences.

The psychosocial impacts of the COVID-19 pandemic have been reported along with various mental health issues in the recent research (90). Although face-to-face interactions were diminished, the exposure to social media and, considering the recruitment procedure of the current study, the use of dating apps increased in this period (91). In a context of social distancing during the pandemic, the evaluation by others could have had an even bigger impact on body image. Additionally, the context could also contribute to increased body surveillance and trigger or aggravate conflicts with oneself regarding body image and lead to negative affect. Thus, it is possible that maladaptive eating attitudes to cope with negative affect might have been intensified during this period. This interpretation is consistent with the interpersonal theory of eating disorders, which views eating disorders as maladaptive coping strategies to regulate emotion in response to negative social interactions (15). Nevertheless, the effects of the pandemic on individuals, especially individuals with sexual minority background, seem not to be fully understood and need further research over the next years.

### Strengths and limitations

The findings of the current study should be interpreted with acknowledgment of its limitations. Since the data collection was online, the sample comprises only of participants with internet access. Considering that one of the recruitment procedures was a dating website, it could shape some of the sociodemographic characteristics of the sample. Not all of the assessments were adequately validated (e.g., Daily Heterosexist Experiences Scale) and the internal consistency of the Brief Resilience Scale was low. In addition, the response characteristics of the participants could not be controlled due to self-report assessments. The majority of the current sample consisted of gay men. It is notable that the other subgroups represent a minority that is still difficult to reach, especially in countries where sexual minorities experience substantial discrimination in the public sphere (89). We understand that social stigma may play an important role when it comes to sexual-minority and gender-diverse populations. There was a certain number of participants who did not complete the survey. We also found differences between the completers and the people who did not complete the online survey. Thus, the characteristics of the sample should be considered when interpreting the findings of the current study. Also, the lack of information on the distribution of sexual minority individuals in Turkey limits the generalizability of the findings. Finally, our ability to infer causal associations between the studied variables was limited due to the cross-sectional study design. Nevertheless, the results of this study are valuable for its input about the LGBTIQ population in Turkey and its ability to stimulate and streamline further research in this field. To our knowledge, the present study was the first to examine eating attitudes, depressive symptoms, and their predictors in a large sample of the LGBTIQ Turkish community. It contributed by identifying disorder-specific psychosocial vulnerability factors and minority stressors, which might inform mental health promotion strategies for this population.

### Future directions

The current findings provide evidence that the minority stressors are significant risk factors beyond the psychosocial vulnerability for eating disorders and depressive symptomatology in this population. Further investigation of direct and indirect relations between the vulnerability factors and the minority stressors could reveal the specific pathways that lead to disturbed eating behaviors. For instance, a previous study showed that an unmet need to belong and perceived stigma predicted increased depression and decreased self-compassion, which in turn were associated with higher levels of disordered eating behaviors among gay men (9). Moreover, longitudinal studies with sexual-minority and gender-diverse populations could investigate the long-lasting impact of the pandemic on their mental health which would show causal associations and risk and protective factors involved. Additionally, further research could focus on sexual minority subgroups about whom there is little literature available.

## Conclusion

The findings demonstrated the significant role of sexual minority stressors in the prediction of disturbed eating attitudes and depression symptoms beyond general psychosocial risk and protective factors. These results emphasize the need to develop strategies to reduce prejudicial attitudes at the societal level and to enhance the skills of LGBTIQ individuals in coping with minority stressors in Turkey.

## Data availability statement

The raw data supporting the conclusions of this article will be made available by the authors, without undue reservation.

## Ethics statement

The studies involving human participants were reviewed and approved by the Research Ethics Committee of Bursa Uludag University. The patients/participants provided their written informed consent to participate in this study.

## Author contributions

HG, TT, and EK designed the study. HG performed the statistical analysis. EK, HG, and ASP drafted the article. ASP implemented the survey. HG, TT, SB, CR-K, and EK discussed the results and contributed to the final manuscript. All authors have approved the final manuscript.

## Funding

This work received funding from the Czech Science Foundation, Project No. 19-27828X. We acknowledge support from Interdisciplinary Research Team on Internet and Society, Faculty of Social Studies, Masaryk University for Open Access Publishing. We also acknowledge support from Open Access Publishing Fund of Leipzig University supported by the German Research Foundation within the program Open Access Publication Funding for Open Access Publishing.

## Conflict of interest

Author CR-K received lecture honoraria from Recordati and Servier, which was outside and independent of the submitted work. The remaining authors declare that the research was conducted in the absence of any commercial or financial relationships that could be construed as a potential conflict of interest.

## Publisher's note

All claims expressed in this article are solely those of the authors and do not necessarily represent those of their affiliated organizations, or those of the publisher, the editors and the reviewers. Any product that may be evaluated in this article, or claim that may be made by its manufacturer, is not guaranteed or endorsed by the publisher.
